# The Impact of Onchocerciasis Elimination Measures in Africa: A Systematic Review

**DOI:** 10.3390/tropicalmed10010007

**Published:** 2024-12-26

**Authors:** Policarpo Ncogo, Christine Giesen, María Jesús Perteguer, Maria P. Rebollo, Rufino Nguema, Agustín Benito, Zaida Herrador

**Affiliations:** 1 Fundación Estatal, Salud, Infancia y Bienestar Social (FCSAI), 28029 Madrid, Spain; pncogo@psglobal.es; 2 Centro de Salud Internacional Madrid Salud, Ayuntamiento de Madrid, 28006 Madrid, Spain; cgiesen@gmx.net; 3 Centro Nacional de Microbiología, Instituto de Salud Carlos III, 28220 Madrid, Spain; chus.perteguer@isciii.es; 4 Consorcio de Investigación Biomédica en Red Enfermedades Infecciosas (CIBERINFEC), 28029 Madrid, Spain; abenito@isciii.es; 5 Expanded Special Project for Elimination of Neglected Tropical Disease (ESPEN), WHO World Health Organization—African Region, P.O.Box 06 Brazzaville, Congo; rebollopolom@who.int; 6 Ministerio de Sanidad y Bienestar Social, Balboa Calle Mayor, Malabo QQ2H+8WC, Equatorial Guinea; rufonguema@yahoo.es; 7 Centro Nacional de Medicina Tropical, Instituto de Salud Carlos III, 28029 Madrid, Spain; 8 Centro Nacional de Epidemiología, Instituto de Salud Carlos III, 28029 Madrid, Spain; 9 Consorcio de Investigación Biomédica en Red Epidemiología y Salud Pública (CIBERESP), 28034 Madrid, Spain

**Keywords:** onchocerciasis, control measures, community directed treatment, Africa

## Abstract

Background: Onchocerciasis, or river blindness, is one of the neglected tropical diseases (NTDs) that the WHO has set out to eliminate. To reach this elimination target, a number of challenges must be met, and the essential measures set out in the road map for NTDs 2021–2030 must be implemented. More than 99% of infected people live in 31 countries in sub-Saharan Africa. Our objective was to assess the impact of onchocerciasis interventions in Africa. Methodology: A systematic peer review of the existing literature following the PRISMA guidelines was performed between November 2021 and April 2022. We selected studies on onchocerciasis control measures in Africa since the implementation of the first Onchocerciasis Control Programme (OCP) measures in 1974. All scientific articles indexed in the PubMed, Scopus, Embase, and CENTRAL databases written in Spanish, English, French, German, and Portuguese were considered. The study protocol was registered in the PROSPERO database. Results: A total of 63 articles met the inclusion criteria and were finally selected. Publications were found from 19 out of 31 African endemic countries. The main intervention retained in the different published studies was mass distribution of ivermectin (*n* = 51). According to our results, 11 African countries have managed to interrupt transmission of onchocerciasis in at least one area in the country; 11 countries have not achieved this goal, while 1 country has managed to eliminate the disease, but it has resurged. Conclusions: Control interventions showed a positive impact on the fight against onchocerciasis, demonstrating that these activities are effective. Nevertheless, they were not sufficient to achieve the proposed goals for a variety of reasons. Therefore, different aspects should be considered in order to fulfil the targets proposed by the WHO to be reached in 2030.

## 1. Introduction

Onchocerciasis is an eye and skin disease and a major public health problem in many parts of the world. The disease is caused by the parasitic worm *Onchocerca volvulus* and transmitted by *Simulium* blackflies. It is endemic in 37 countries in West, East and Central Africa; the Arabian Peninsula; and parts of South and Central America. More than 99% of infected people live in 31 countries in sub-Saharan Africa. The Global Burden of Disease Study estimated in 2017 that at least 220 million people required preventive chemotherapy against onchocerciasis, 14.6 million of the infected people already had skin disease, and 1.15 million had vision loss [1].

Between 1974 and 2002, onchocerciasis was brought under control in West Africa through the work of the Onchocerciasis Control Programme (OCP). This programme relied mainly on the spraying of insecticides against black fly larvae (vector control) from helicopters and aeroplanes. Lately, these activities were complemented by the donation and large-scale community-based distribution of ivermectin (CDTI) starting in 1989. This drug (registered for human use as Mectizan^®^) was donated by Merck & Co Inc. (Rahway, NJ, USA) in 1987. The OCP was a paradigmatic successful programme in the control of tropical diseases in general and onchocerciasis in particular [2]. According to WHO estimates, it has prevented the infection of 40 million people, prevented 600,000 people from blindness, and allowed 18 million children to be born free from the threat of this disease [3]. Nevertheless, while the combination of large-scale larvicide OCP operations plus mass ivermectin treatment were eliminating the disease as a public health problem in some African countries, other endemic countries outside the OCP were stuck in their fight [2,4]. For this reason, the African Programme for Onchocerciasis Control (APOC) was launched in 1995 to develop self-sustainable ivermectin treatment programmes in highly endemic areas of 19 countries in Africa. In the last year of APOC (2015), more than 119 million people were treated with ivermectin, and many countries achieved a large reduction in morbidity rates associated with onchocerciasis [4]. However, the good results of both programmes were clouded in the areas where *O. volvulus* and *Loa loa* coexist due to the appearance of cases of encephalopathies in co-infected patients [5].

These events, together with the paradigm shift from control to elimination, promulgated the creation of the Expanded Special Project for the Elimination of Neglected Tropical Diseases (ESPEN). This project was established in May 2016 as a public–private partnership between the WHO Regional Office for Africa (AFRO), the Member States, and NTD partners to mobilise policies, techniques, and financial resources to accelerate elimination of the five most prevalent NTDs, which include onchocerciasis [6,7]. In addition, in 2017 the WHO created the Onchocerciasis Technical Advisory Subgroup, whose main role is to make recommendations on future policy, guideline development and research priorities to help the programme achievements towards onchocerciasis elimination [7,8].

To continue moving forward towards onchocerciasis elimination, it is necessary to assess the different strategies that have been implemented during the last 48 years, as well as their impact, and the pending challenges that remain in areas where the disease persists. For this reason, we have carried out a systematic review to evaluate the impact of onchocerciasis elimination activities in sub-Saharan Africa, i.e., increases or decreases in the presence of onchocerciasis cases and onchocerciasis vectors, from the creation of the OCP to the present.

## 2. Materials and Methods

We performed a systematic peer review following the Preferred Reporting Items for Systematic Reviews and Meta-Analyses (PRISMA) guidelines between November 2021 and April 2022 [9]. The PRISMA checklist can be accessed in Supplementary Material. The protocol was registered in PROSPERO (University of York, York, UK) and accepted in April 2021 (ID CRD42021236833) (https://www.crd.york.ac.uk/PROSPERO/, (accessed on 20 October 2021).

In November 2021 we searched the PubMed, Scopus, Embase, and CENTRAL databases for all journal articles published since 1974, in Spanish, English, French, German, and Portuguese by using three sets of terms: the components of the disease and its transmission (onchocerciasis and *Simulium* spp.); control measures; and Africa.

Our study area was the African region and specifically those countries considered endemic for onchocerciasis, currently or in the past. We set the inclusion year based on the launch of the OCP in West Africa in seven countries (Benin, Burkina Faso, Côte d’Ivoire, Ghana, Mali, Niger, and Togo), which focused on vector control and then continued with the APOC in 1995 [10]. We used the following search strategy:

(“Onchocerciasis” OR “river blindness” OR “mutumutu” OR “*Simulium damnosum*” OR “*Simulium neavei*” OR “*Simulium ochraceum*” OR “*Simulium*” OR “simuliidae” OR “simulid” OR “black fly” OR “blackfly”) AND (“treatment with ivermectin” OR “treatment with ivermectin under community directives” OR “Community-directed treatment with ivermectin” OR “CDTI” OR “ivermectin” OR “vector control” OR “larvicide control” OR “ground fumigation” OR “aerial fumigation” OR “fumigation” OR “control”) AND (“Africa” OR “Angola” OR “Burundi” OR “Cameroon” OR “Central African Republic” OR “Chad” OR “Democratic Republic of the Congo” OR “Congo” OR “Ethiopia” OR “Equatorial Guinea” OR “Gabon” OR “Kenya” OR “Liberia” OR “Malawi” OR “Mozambique” OR “Nigeria” OR “Rwanda” OR “Sudan” OR “South Sudan” OR “Tanzania” OR “Uganda” OR “Senegal” OR “Mali” OR “Gambia” OR “Burkina Faso” OR “Guinea Bissau” OR “Guinea” OR “Sierra Leone” OR “Ghana” OR “Benin” OR “Cote d’Ivoire” OR “Ivory Coast” OR “Togo”).

We included descriptive, cross-sectional, longitudinal, ecological, or analytical articles targeting onchocerciasis control measures in the Africa region. Our inclusion and exclusion criteria were as follows.

### Inclusion and Exclusion Criteria

Descriptive, longitudinal, or cross-sectional studies addressing human onchocerciasis or its vectors or analysing control measures for this disease performed in the African continent published since 1974 were eligible for inclusion.

We excluded studies addressing other infectious diseases or those performed outside the African continent, as well as articles published in languages other than Spanish, French, Portuguese, English, or German, or using study designs other than those mentioned above.

Two independent reviewers read the abstracts and full articles to decide whether the selected papers met the inclusion criteria. In case of disparities, a third reviewer was asked for a blind decision.

Data were collected pertaining to the disease, implemented control measures, and the specific African regions where the study was conducted. Tables presenting evidence were constructed for this purpose. The most pertinent findings were documented based on the implemented control measures, the level of evidence, and the study limitations. Variables collected included the type of onchocerciasis disease, the type of vector, geographical location, methodology, implemented control measures, primary outcomes, as well as the level of evidence and limitations. Mixed intervention is understood as the implementation of two onchocerciasis control strategies, like the combination of CDTI and vector control [11]. A tool for assessing study quality, derived from similar literature reviews [12,13,14,15], was used. This tool evaluated content, study design, and result presentation, awarding 1 point for compliance and 0 for non-compliance. The maximum attainable score was 12 points.

We used Excel and Zotero to record and summarise the results. A database was created in Excel to collect the data. Zotero (Version 5.0.67, Corporation for Digital Scholarship, Vienna, VA, USA; www.zotero.org, accessed on 21 October 2021) was used as a bibliographic reference search engine. Additionally, the study selection process was performed in the systematic review tool Rayyan (Cambridge, MA 02142 USA). Mapchart (Version 2023, https://www.mapchart.net/, accessed on 6 June 2022) and Canva (Version 2023, Sydney, Australia; www. https://www.canva.com/, accessed on 20 June 2022) were used to create maps and explanatory figures.

## 3. Results

### 3.1. Search Results

**Figure 1:**
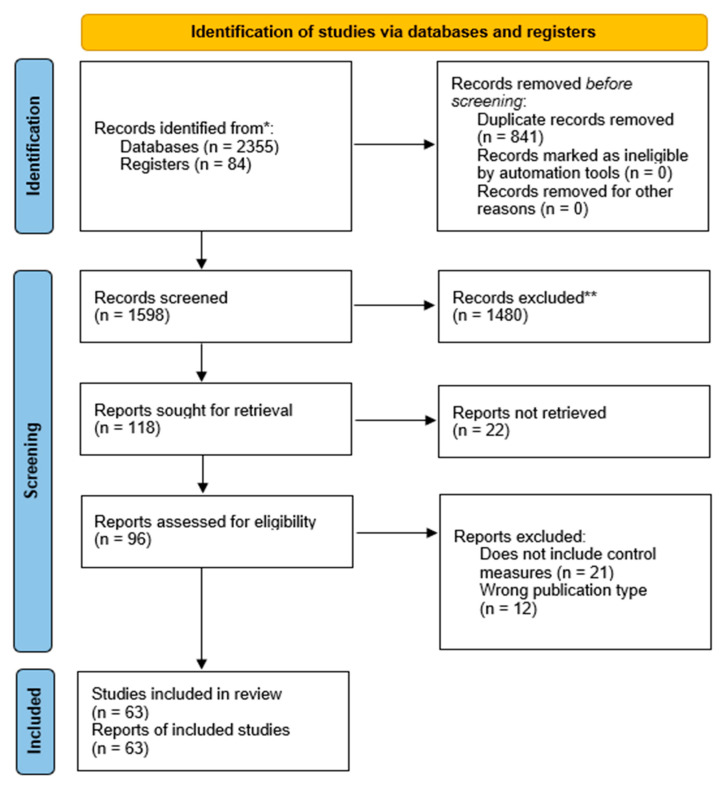
PRISMA flow chart. * Consulted databases and registers were PubMed, Scopus, Embase, and CENTRAL. ** No automation tools were used.

We identified 2355 articles in the search, which was performed from November 2021 until February 2022. After the first abstract and title filtering, 118 articles were selected for full-text reading, out of which 63 articles met the inclusion criteria and thus were finally included (Figure 1).

**Figure 2:**
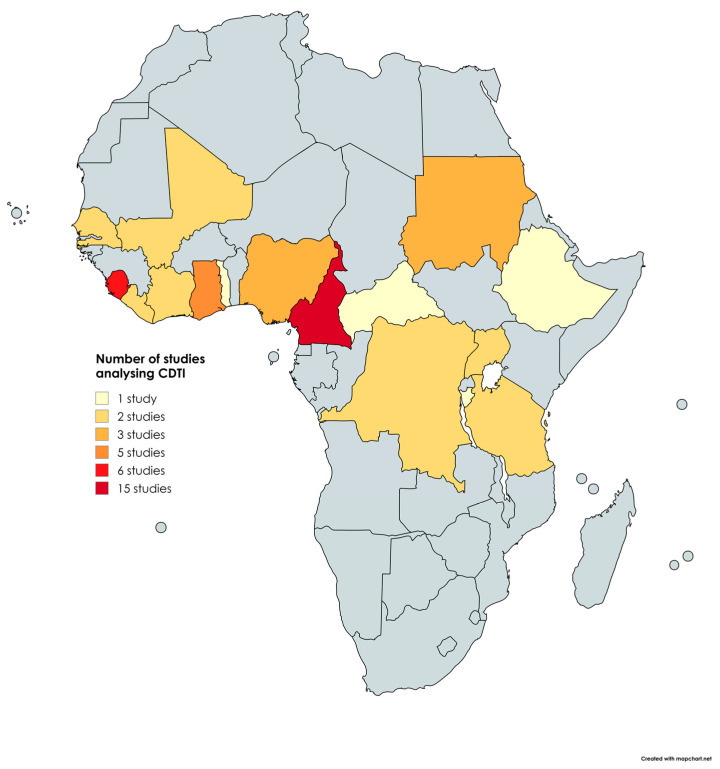

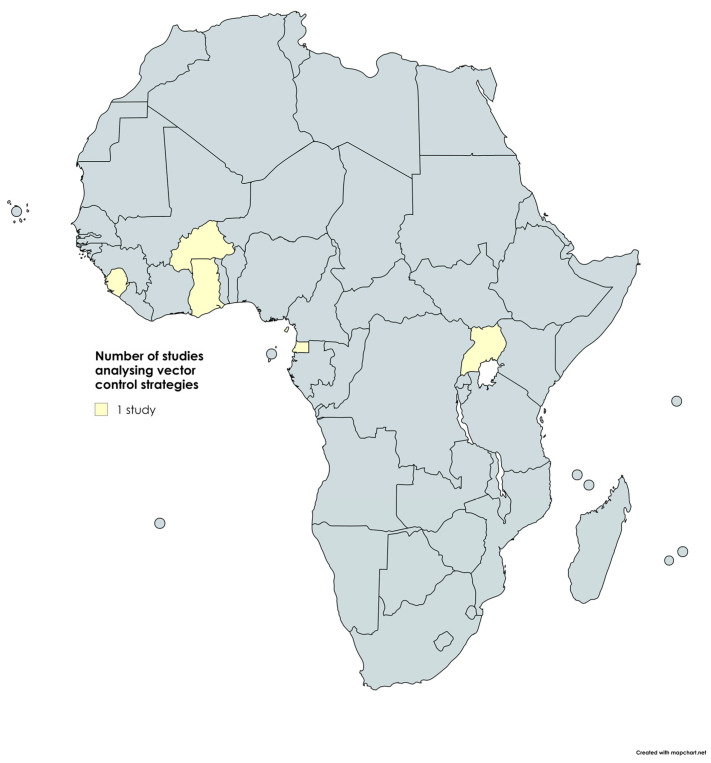

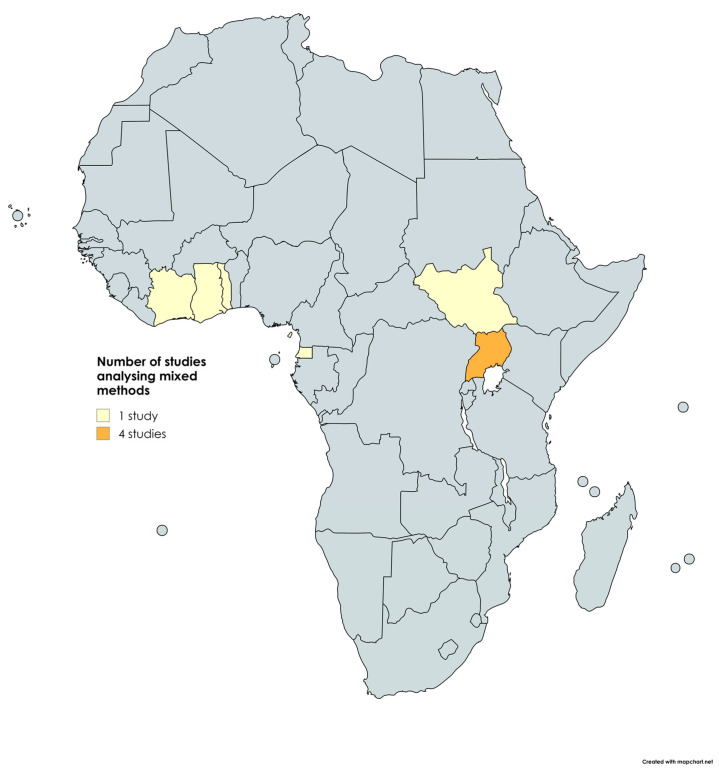
Published articles by African endemic countries. **Above**: Countries with studies assessing only CDTI. **Middle**: Countries assessing only vector control strategies. **Below**: Countries assessing mixed methods. Countries where no studies were performed are represented in grey.

The 63 included papers were published in 19 of the 31 countries considered endemic for onchocerciasis in the Africa region [1] were published from 1995 onwards. The distribution by country of study is shown in Figure 2.

CDTI was used in 17 countries (89.5%) with a total of 51 (81.0%) reviewed articles [5,10,16,17,18,19,20,21,22,23,24,25,26,27,28,29,30,31,32,33,34,35,36,37,38,39,40,41,42,43,44,45,46,47,48,49,50,51,52,53,54,55,56,57,58,59,60,61], while mixed intervention was assessed in 6 countries (31.6%), with 9 (14.3%) reviewed articles [61,62,63,64,65,66,67,68,69]. Vector control was analysed as the only strategy for onchocerciasis elimination in 5 articles performed in 5 countries [11,38,70,71,72], i.e., ground spraying and insecticide against blackfly larvae (Table 1).

Of these studies, 22 articles were published in 8 countries where interruption of transmission has been demonstrated in specific areas or zones: Uganda [38,58,59,64,65,66,69,75,76,77], Sudan [36,53,54], Nigeria [46,47,60], Mali [44,45], Senegal [44,45], Equatorial Guinea [11,67], Burkina Faso [70,73], and Ethiopia [36], although in the case of Burkina Faso, the disease reappeared [70,73]. In these eight countries, ivermectin distribution started in the 1990s, except in the areas of Metema (Ethiopia) and Galabat (Sudan) [36], where it began in 2003. The duration of ivermectin treatment ranged from 10 years in the Galabat area of Sudan [36] to 26 years in the areas of Bioko (Equatorial Guinea) [11] and Plateau and Nasarawa (Nigeria) [60]. The average mean of minimum and maximum years to demonstrate the interruption of the transmission in certain areas of these 8 countries were 15 (standard deviation (SD) = 4.34) and 21 (SD = 5.05). Nevertheless, there was a high disparity in the type of assessment applied in each country (Table 2).

**Figure 3:**
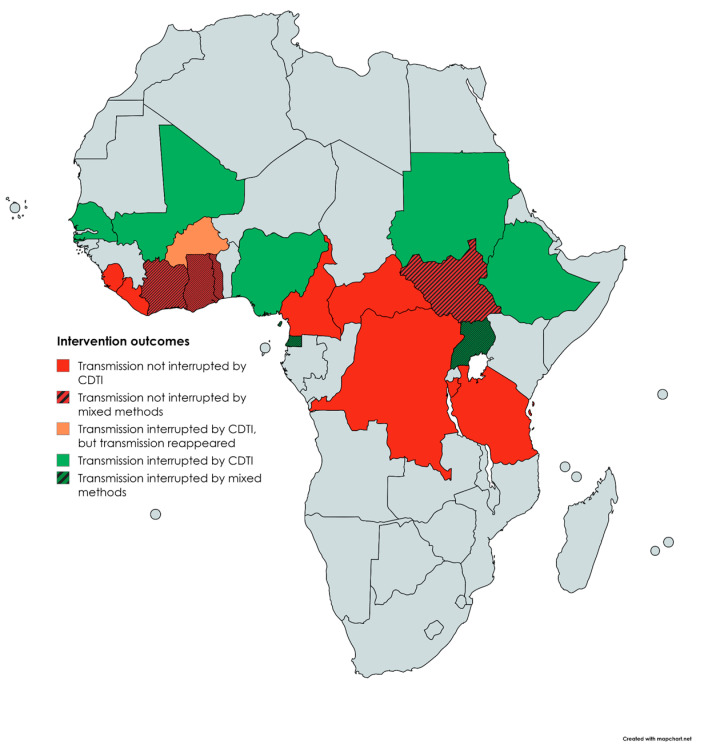
Intervention outcomes by country. Some studies were performed in certain regions of the countries. However the whole country shape is highlighted for better visibility. Countries where no studies were performed are represented in grey.

In addition to the mass distribution of ivermectin, vector control interventions (ground and aerial larvicides) were also implemented in 7 out of these 8 countries: Uganda [38,65,66,69,76,77], Equatorial Guinea [11,67], Burkina Faso [70], Ghana [62,71], Côte d’Ivoire [61], Togo [63], and Sierra Leone [72]. A summary of the main results by country is provided below. Figure 3 shows the outcomes by country.

### 3.2. Results by Country

#### 3.2.1. Countries That Have Successfully Interrupted Transmission of Onchocerciasis in Certain Areas (Table 3 and Table 4)

##### Uganda

Of the 17 endemic foci of onchocerciasis in Uganda, 9 have interrupted onchocerciasis transmission [38,58,59,64,65,66,69,75,76,77]. Mass distribution of ivermectin was used in the Imaramagambo [59], Wadelai [75], and Obongi [58] regions, while in the Wambabya–Rwamarongo [65], Kashoya–Kitomi [64], Mount Elgon [66], Itwara [69], and Mpamba Kusi areas [77], a mixed intervention was used. Only in the Victoria Nile was the focus vector treatment used under the OCP [76].

The duration of mass distribution of ivermectin lasted a minimum of 14 years in the Wadelai focus [75] and a maximum of 24 years in Wambabya–Rwamarongo [65]. Parasitological, entomological, and serological assessments were carried out in the different foci. These evaluations demonstrated the interruption of onchocerciasis in 9 out of the 17 foci [38,58,59,64,65,66,69,75,76,77]. The Victoria Nile area was declared free of onchocerciasis in the early 1970s, using fumigation with dichlorodiphenyltrichloroethane (DDT) as the main intervention, but it was not confirmed according to the current WHO verification guidelines until 2020, bringing it in line with other more recently eliminated foci [76]. On the other hand, a study conducted by Ndyomugyeny et al. in Uganda in 2002 and published in 2004 showed that ivermectin treatment in parallel to vector elimination had a greater impact on dermatitis and microfilariae carrier rates than ivermectin treatment alone [78].

##### Nigeria

In Nigeria, the CDTI was first implemented in 3 known endemic foci [46,47,60]. These foci were located in Plateau, Nasarawa [60], Adani [47], and Kaduna [46] regions. In Plateau and Nasarawa State, ivermectin distribution lasted between 8 and 26 years, starting in 1992 and ending in 2017 in the case of Plateau and Nasarawa [60] ; from 1991 to 2008 in Kaduna [46]; and from 1991 to 2008 in the Adani region [47]. Several entomological [47,60] and serological [60] and parasitological [46] evaluations have been carried out, demonstrating the interruption of onchocerciasis transmission in these four states [46,47,60].

##### Mali

Two studies were conducted in Mali. In the onchocerciasis hotspots, located along the Bakoya River, mass distribution of ivermectin began under the umbrella of OCP between 1988 and 1989, lasting between 15 and 17 years [44,45]. Evaluation through parasitological and entomological surveys by Traoré et al. (started in 2006 and completed in 2011) showed that infection and transmission levels continued to decline, as had been previously shown during ivermectin treatment, and 3–4 years after the last year of CDTI both indicators were zero [44].

##### Senegal

Two studies were conducted in Senegal, where CDTI was launched between 1988 and 1989 by OCP at six-month intervals, instead of annually, along the Gambia River [44,45]. The results of entomological and parasitological assessments carried out between 2006 and 2011 by Traoré et al. threw positive results, and 3–4 years after the last round of CDTI, no microfilariae (mf) or infective larvae were detected. In the case of the Faleme River (at the Senegal/Mali border, with annual ivermectin administration), despite observing comparatively high infection levels before ceasing treatment, there was a consistent decline in infection and transmission to very low levels 3 to 5 years after treatment was stopped [44].

##### Sudan

Three studies were performed in Sudan [36,53,54]. In the Abu Hamed region, the northernmost onchocerciasis site in the world, CDTI distribution was annual from 1998 to 2007 and biannual thereafter [53,54]. In the Galabat region, CDTI started at the end of 2007. From 2011, distribution was increased to twice a year until 2014, when it reverted to annual treatment. In the Galabat region, onchocerciasis control was coordinated with the Metema border area of Ethiopia [36].

In 2011, 13 years after the start of treatment, the entomological, parasitological, and transmission statuses were assessed in the Abu Hamed region, and the results of the study conducted by Higazi TB et al. demonstrated the interruption of onchocerciasis transmission [53]. Due to these good results, treatment was stopped in 2012, and a 3-year post-treatment surveillance was initiated, at the end of which an evaluation was conducted in 2015 following WHO guidelines. The results of this evaluation showed for the first-time verification of onchocerciasis elimination following successful post-treatment surveillance in Africa [54].

##### Equatorial Guinea

In Equatorial Guinea, with 2 published studies, ivermectin was distributed by mobile teams from 1990 to 1997 on the island of Bioko [11,67]. Starting from 1998, it was changed to CDTI within the framework of the APOC Programme [11]. Between 2001 and 2005, a large-scale larvicide (Temephos) trial was carried out with ground and aerial applications. In 2005, the endemic vector form of Bioko (*S. yahense*) was finally eliminated from the island [67]. According to personal information from staff of the Ministry of Health and Social Welfare of Equatorial Guinea (MINSABS), the last CDTI round was administered in 2012 in the urban area of Malabo and in 2016 in the rest of the island. Evaluations conducted to measure the impact of these interventions through entomological and serological surveys demonstrated the interruption of onchocerciasis transmission in Bioko Island, resulting in the interruption of the CDTI and the implementation of post-treatment surveillance to identify any new cases of exposure or infection in Bioko island [67].

##### Ethiopia

The annual CDTI was launched in the Metema region in 2003 and continued until 2016, when a six-month treatment was initiated. Treatments were also completed in this focus in 2017 in coordination with the Galabat sub-focus. The results of the study conducted by Katabarwa MN et al. in the Metema and Galabat foci in Ethiopia and Sudan have shown interruption of onchocerciasis transmission [36].

**Table 3:** Countries that have successfully interrupted transmission of onchocerciasis in certain areas. The countries are listed in the table according to the number of articles published from highest to lowest. (ND: No data).

Country	Author	Year of Publication	Applied Intervention		Regions with Transmission Interruption
			CDTI (Yes/No)/Start Date (Year)/Duration in Years	Vector Control (Yes/No)/Start Date (Year)/Duration in Years	
Uganda	Garms R [38]	2009	No	Yes/1995/8	Itwara
	Katabarwa MN [75]	2012	Yes/1993/14	No	Wadelai
	Lakwo TL [69]	2013	Yes/1991/19	Yes/1995/ND	Itwara
	Katabarwa MN [66]	2014	Yes/1994/17	Yes/2008/1	Mount Elgon
	Lakwo TL [77]	2015	Yes/1995/17	Yes/2002/ND	Mpamba Kusi
	Katabarwa MN [59]	2016	Yes/1993/17	No	Imaramagambo
	Lakwo T [64]	2017	Yes/1991/19	Yes/2003/3	Kashoya–Kitomi
	Luroni LT [58]	2017	Yes/1993/20	No	Obongi
	Katabarwa MN [65]	2020	Yes/2008/5	Yes/2009/ND	Wambabya–Rwamarongo
	Katabarwa MN [76]	2020	No	Yes/1951/22	Nile Victoria
Nigeria	Tekle AH [46]	2012	Yes/1991/17	No	Kaduna
	Richards FO [60]	2020	Yes/1992/26	No	Plateau and Nasarawa
	Onah IE [47]	2020	Yes/1996/15	No	Adani
Sudan	Higazi TB [53]	2013	Yes/1998/14	No	Abu Hamed
	Zarroug IM [54]	2016	Yes/1998/14	No	Abu Hamed
	Katabarwa MN [36]	2020	Yes/2007/11	No	Galabat
Mali	Diawara L [45]	2009	Yes/1998/17	No	Bakoye and Faleme
	Traore O [44]	2012	Yes/1998/17	No	Bakoye and Faleme
Senegal	Diawara L [45]	2009	Yes/1998/17	No	Gambia and Faleme
	Traore MO [44]	2012	Yes/1998/17	No	Gambia and Faleme
Equatorial Guinea	Mas J [11]	2006	Yes/1990/26	Yes/2001/4	Bioko Island
	Cheke RA [79]	2009	No	Yes/2001/Week	Bioko Island
	Herrador Z [67]	2009	No	Yes/2001/4	Bioko Island
Ethiopia	Katabarwa MN [36]	2020	Yes/2003/14	No	Metema

**Table 4:** Type of assessment and results in countries that have successfully interrupted transmission of onchocerciasis in certain areas. The countries are listed in the table according to the number of articles published, from highest to lowest. OV16: diagnostic tests based on ELISA detection of antibodies to the OV16 antigen.

Country	Author	Year of Publication	Year of Evaluation	What Kind of Evaluation	Population Assessed	Result of the Evaluation
Uganda	Garms R [38]	2009	2003	Entomological	Flies	0 flies found
	Katabarwa MN [75]	2012	2009	Parasitological	500 people	0 microfilariae
			2009	Serological	3011 children	1 child (+) OV16
	Lakwo TL [69]	2013	2010 & 2012	Serological	3316 children	2 (+) OV16. In 2012, 21 months later the 2 children were (−) OV16.
				Parasitological	688 skin snips	No evidence of any microfilaria
	Katabarwa MN [66]	2014	2009	Serological	3051 children	1 child (+)
			2015	Entomological	Crabs and flies	Infestation reduced from 41.9% 2007 to 0% and 0 fly bites
	Lakwo TL [77]	2015	2008	Entomological	14,391	0 infested
			2009 & 2012	Serological	3351 <15 years and 3407	0.6% while in 2012 only 1 out of 3407 (+)
			2012	Parasitological	732 people	0.3% of microfilariae
	Katabarwa MN [59]	2016	2015	Entomological	Fly Catching	0 flies caught since 2007
	Lakwo T [64]	2017	2004 & 2013	Parasitological	Number of microfilariae	From 85% to 62% in 2004 to 0.5% 2013
			2010	Serological	1362 children	11 children (+) OV16
			2009 & 2013	Entomological	Crabs and flies	Crab infestation reduced from 59% to 0%. Fly Catch from 5334 to 0
			2012	Serological	3246 children	5 children (+) OV16 and 4 children out of 5 (−) PCR
	Luroni LT [58]	2017	2012	Parasitological	807 people	0% microfilariae
			2013	Serological	3308 <10 years	3 (+) OV16, by PCR all 3 (−)
	Katabarwa MN [65]	2020	2013	Serological	2978 minors	1 positive only
			2016	Entomological	10578 crabs	0 crabs infested, 0 fly caught
			2017	Serological	3079 minors	0 positive
	Katabarwa MN [76]	2020	ND	Entomological	854 flies	0 infected
			ND	Serological	2953 children	All (−) OV16
Nigeria	Tekle AH [46]	2012	2008	Parasitological	3703 >1 year	0% prevalence
	Richards FO [60]	2020	2017	Serological	6262 <10 years	2 children (+)
			2017	Entomological	19,056 flies	None infested
	Onah IE [47]	2020	2011	Entomological	548 flies	0 infested
Sudan	Higazi TB [53]	2013	2011	Parasitological	536 persons	Skin microfilariae were absent
			2011	Serological	6756 <10 years	No evidence of *O. volvulus* antibodies was found
			2011	Entomological	17,536 flies	O-150 pool screening showed no parasite DNA in 17,537 black flies (95% CI UL 0.023)
	Zarroug IM [54]	2016	2015	Serological	5266 children	Only one Ov16 seropositive child (0.019%, 95% UCL = 0.074); whose skin snips were negative when tested by O-150 PCR assay
			2015	Entomological	19,266 flies	Polymerase chain reaction (PCR) screening of 19,191 flies from four sites for the O-150 parasite-specific marker found no flies carrying *O. volvulus* larvae (0%, 95% upper confidence limit (UCL) = 0.16)
	Katabarwa, M.N [36]	2020	2014 & 2015	Serological	6072 y 3931 <10 years	8 (+) OV16 and (−) PCR
			2014 & 2016	Entomological	27,583 and 9148 flies	0.14 of infective flies
Equarorial Guinea	Mas J [11]	2006	1998	Parasitological	1082 people	The crude *O. volvulus* infection prevalence before ivermectin intervention was 74.5% (1284/1723); after the intervention it was 38.4% (415/1082).
	Cheke R [79]	2009	ND	Entomological	Flies	0 flies caught
	Herrador Z [67]	2018	2016	Entomological	Fly Capture	All fly collections and larval prospections in the traditional catching and prospection sites were negative.
			2016	Serological	7052 <10 years	4 (+) OV16 and (−) ELISA
Mali	Diawara L [45]	2009	ND	Parasitological	17,801	Prevalence < 1%
					2283 people	
			ND	Entomological	157,500 flies	Infestation rate 0.5
					123,000 flies	
	Traore MO [44]	2012	2006 & 2011	Parasitological	29,753 people	Indicators below the elimination threshold
			2006 & 2011	Entomological	492,600 flies	Indicators below the elimination threshold
Senegal	Diawara L [45]	2009	ND	Parasitological	17,801/2283 people	Prevalence < 1%
					2283 people	
			ND	Entomological	157,500 flies	Infestation rate 0.5
					123,000 flies	
	Traore MO [44]	2012	2006 & 2011	Parasitological	29,753 people	Indicators below the elimination threshold
			2006 & 2011	Entomological	492,600 flies	Indicators below the elimination threshold
Ethiopia	Katabarwa MN [36]	2020	2014 & 2015	Serological	6072 and 3931 <10 years old	8 (+) OV16 and (−) PCR
			2014 & 2016	Entomological	27,583 and 9148 flies	0.14 of infective flies

ND: No data; OV16: Onchocerca polymorphism diagnostic tests based on antibody detection by ELISA OV16; PCR: Polymerase chain reaction.

#### 3.2.2. Countries That Have Failed to Interrupt the Transmission of Onchocerciasis (Table 5 and Table 6)

##### Cameroon

Cameroon was the country with the most published studies during the study period; a total of 15 [5,17,18,19,20,21,22,23,24,25,26,27,28,29,30]. Mass treatment with ivermectin was initiated in 1987 [17]. However, to date, all parasitological and entomological assessments indicate that transmission is still ongoing [5,17,19,20,21,22,23,24,25,26,27,29,30]. The occurrence of severe and even fatal side-effects after treatment due to the presence of loasis may have played a role in this. This was the case of the region in the Okola health district, where ivermectin distribution was stopped in 1999 after the occurrence of fatal events related to L. loa [23]. Furthermore, in 2021, a study conducted by Abong RA showed that the dense river system of the Mbam drainage generated important breeding sites for the vector larvae, leading to an abundance of *Simulium* spp. in both the dry and rainy seasons, thus favouring transmission of the disease [22].

##### Sierra Leone

Seven studies from Sierra Leone were retained in this review [10,48,49,50,51,52,72]. According to Koroma et al., the National Onchocerciasis Control Programme (NOCP) was established in 1989 under the OCP, with vector measures being the only intervention used at that time [10]. However, the civil conflict between 1991 and 2002 had a negative impact on onchocerciasis control activities [45]. Thus, between 1997 and 2002, only limited activities were carried out in high prevalence areas and with security; therefore, treatment coverage before 2002 was deemed not reliable. Interventions were restarted in 2003 under the APOC, with CDTI as the main strategy [10]. Parasitological [10,48,49,51], entomological [52,72], and ocular [50] assessments conducted between 2005 and 2009 have shown a significant reduction in the prevalence and mean density of onchocerciasis in the 12 endemic districts of Sierra Leone but not in the discontinuation of transmission [10].

##### Ghana

Seven studies were carried out in Ghana. The OCP started in 1974, mainly based on the weekly aerial spraying of rivers, where vector species of the *S. damnosum* complex were breeding, with the insecticide Temephos. The annual mass distribution of ivermectin started in 1987 [37,39,40,41,62,71,74]. Several entomological and parasitological evaluations were conducted to assess the impact of these interventions [37,39,40,41,62,71,74]. In 2007, Osei-Atweneboana and co-workers reported on the epidemiological situation of onchocerciasis in Ghana after the closure of the OCP and observed that, despite vector control and 19 years of annual ivermectin treatment, some communities showed a high prevalence of onchocerciasis [41]. In the same line, in 2019, a study conducted in selected hypo-endemic communities following long-term administration of ivermectin showed that despite several years of mass administration, onchocerciasis infection, and clinical manifestations commonly associated with the disease still persisted [40].

In Ghana, two brands of commercially available repellents were also tested for use: N, N-diethyl-3 methylbenzamide (DEET) with active concentrations of 9.5%, 13%, 25%, 50%, and 98.1–100% and “NO MAS” (active component: Para-menthane-3,8-diol and lemongrass oil). NO MAS was more effective, with 5 h of effectiveness versus DEET with 1 h of effectiveness [71].

##### Côte d’Ivoire

In Côte d’Ivoire, the fight against onchocerciasis started with aerial insecticide application in 1974 [32,33,61]. From 1992 to 2018, most of the endemic areas had been covered by CDTI [61]. Although the country experienced periods of civil unrest that interrupted CDTI for several years, significant progress towards onchocerciasis elimination was made during this time. Parasitological assessments carried out have revealed significant reductions in both mf prevalence and mf loads in the communities studied [32,33,61]; such is the case of the assessment carried out by Koudou BG et al. in 2018 [61].

##### Togo

The northern territories of Togo were part of the initial vector control intervention zones of the OCP (1976), while the central regions were included in 1987. In 1988, vector treatment was complemented by CDTI [57,63]. After more than two decades of interventions, parasitological, entomological, serological [63], and ocular assessments [57,63] carried out in these foci showed a significant reduction in transmission. However, the geographic and demographic conditions of the Oti, Keran, and Mov river basins seem to require continued, comprehensive, intensified, and well-adapted interventions that should go beyond mass distribution of Ivermectin [63].

##### Liberia

In Liberia, CDTI has been the main intervention implemented in Bassa County, starting in 1987 [42,43]. The only published evaluation dating from 1991 showed that CDTI is safe, well accepted, and effective in reducing the mf burden [43].

##### Democratic Republic of Congo (DRC)

In the DRC, the CDTI was implemented in 2001 in the Kasai area and has been progressively extended to the other endemic areas of the country. However, after more than 10 years of intervention and according to the published evaluations, the DRC has not succeeded in interrupting onchocerciasis transmission for various reasons, such as fear of serious side effects, non-distribution of the drug due to insecurity and inaccessibility, among others [34,35].

##### Tanzania

In Tanzania, CDTI was implemented in 1997, and vector control strategies between 2003 and 2005, although in some areas the community treatment started in 1994. Parasitological [55] and entomological [56] assessments in the Tukuyu and Mahenge region have shown a significant impact on reducing disease transmission [55,56].

##### Central African Republic

In the Gami focus, mass distribution of ivermectin has been used since 1990. After several successful parasitological evaluations in 1995, 1998 and 2002, Yaya et al. conducted another parasitological evaluation in 2010 to assess the evolution of the prevalence of onchocerciasis after 20 years of mass treatment. The results of this evaluation suggest that onchocerciasis has been controlled rather than eliminated in the Gami villages because of the persistence of a significant prevalence of microfilariae [31].

##### Burundi

In November 1990, mass distribution of ivermectin began in Burundi. Although it was called mass distribution, the compliance in this population was incomplete and far from regular. In June 1994, a parasitological evaluation was carried out. The results indicated that even irregular attendance at ivermectin mass distribution sessions can affect onchocerciasis transmission and significantly reduce the microfilariae load in the affected communities [16].

##### South Sudan

The main control strategy for *O. volvulus* was CDTI, which was complemented by a community-based vector control method called “slash and clear” at the Maridi dam, a breeding site for *S. damnosum* s.l. The results of this study showed a >90% decrease in biting rates of *S. damnosum* s.l. close to the Maridi dam after applying “slash and clear” for more than six months. Twelve months after this intervention, the reduction in biting rates was still at <50% (*p* = 0.0007). Parity rates reduced from 13% pre-“slash and clear” (November 2019) to 5.6% post-“slash and clear” (November 2020) [68].

Table 5 shows countries where transmission has not been interrupted. Table 5 shows the type of assessment and results in these countries. The countries are listed in the table according to the number of articles published, from highest to lowest. See attached.

**Table 5:** Type of assessment and results in countries where transmission has not been interrupted. The countries are listed in the table according to the number of articles published, from highest to lowest.

Country	Author	Year of Publication	Applied Intervention		Main Reasons for Maintained Transmission
			CDTI (Yes/No)/Start Date (Year)/Duration in Years	Vector Control (Yes/No)/Start Date (Year)/Duration in Years	
Cameroon	Gardon J [26]	1997	Yes/ND/ND	No	Occurrence of severe side effects in an endemic area with Loa loa, after treatment.
	Boussinesq M [5]	1997	Yes/1987/8	No	Unknown reasons for not fully understanding the epidemiological significance of ongoing transmission.
	Seidenfaden R [27]	2001	Yes/1987/10	No	Unknown reasons for not fully understanding the epidemiological significance of ongoing transmission.
	Twum Danso N [24]	2003	Yes/ND/ND	No	Occurrence of serious side effects in an endemic area with Loa loa.
	Pion SD [30]	2004	Yes/1991/4	No	Low coverage.
	Kamga HL [18]	2011	Yes/2004/6	No	Mid-term evaluation.
	Richards FO [28]	2011	Yes/1992/17	No	Unknown reasons for not fully understanding the epidemiological significance of ongoing transmission.
	Katabarwa MN [25]	2013	Yes/1996/15	No	Unknown reasons for not fully understanding the epidemiological significance of ongoing transmission.
	Wanji S [29]	2015	Yes/1989/10	No	Onchocerciasis endemic area and *Loa loa* and ecological factors strongly favour onchocerciasis transmission.
	Eisenbarth A [17]	2016	Yes/1987/25	No	This study may reveal the impact of the presence of animal filariasis on the transmission of onchocerciasis.
	Kamga GR [19]	2016	Yes/1999/15–16	No	possible coverage issues and socio-anthropological considerations related to the CDTI.
	Kamgno J [23]	2017	Yes/ND/ND	No	Interruption of ivermectin treatment since 1999 due to severe side effects.
	Siewe Fodjo JN [20]	2018	Yes/1990/13	No	Sub-optimally implemented control measures.
	Boullé C [21]	2019	Yes/1998/19	No	Unknown reasons for not fully understanding the epidemiological significance of ongoing transmission.
	Abong RA [22]	2021	Yes/1997/20	No	The possible fluvial system of the Mbam river drainage generates important breeding sites, which causes abundant *Simulium* in both the dry and rainy seasons.
Sierra Leone	Whitworth JAG [50]	1991	Yes/ND/2	No	Unknown reasons for not fully understanding the epidemiological significance of ongoing transmission.
	Njoo FL [48]	1992	Yes/1987/2	No	Unknown reasons for not fully understanding the epidemiological significance of ongoing transmission.
	Bissan Y [72]	1995	No	Yes/1989/5	Unknown reasons for not fully understanding the epidemiological significance of ongoing transmission.
	Chavasse DC [52]	1995	Yes/1987/3	No	Unknown reasons for not fully understanding the epidemiological significance of ongoing transmission.
	Whitworth JAG [49]	1996	Yes/1987/6,5	No	Unknown reasons for not fully understanding the epidemiological significance of ongoing transmission.
	Kläger SL [51]	2007	Yes/1987/6	No	Unknown reasons for not fully understanding the epidemiological significance of ongoing transmission.
	Koroma JB [10]	2018	Yes/2005/ND	No	Unknown reasons for not fully understanding the epidemiological significance of ongoing transmission.
Ghana	Alley EN [39]	1994	Yes/1987/5	No	It is a part-time evaluation.
	Osei-Atweneboana MY [41]	2007	Yes/ND/4	No	The microfilaricide effect of Ivermectin, but that adult populations of resistant and unresponsive parasites are emerging.
	Wilson MD [71]	2012	No	Yes/ND/ND	The effect of different types of repellents for the prevention of *Simulium* damnosum bites is tested.
	Lamberton PHL [62]	2015	Yes/1987/24	Yes/1975/27	Unknown reasons for not fully understanding the epidemiological significance of ongoing transmission.
	Garms R [74]	2015	Yes/1999/13	No	Intermittent disappearance of the vector.
	Agyemang ANO [37]	2018	Yes/1999/13	No	Treatment adherence rates decreased for the following reasons: fear of unpleasant side effects, lack of awareness of ITDPs, participants believed that treatments were no longer necessary due to the absence of the vectors observed in 2013, motivation of the distributors.
	Otabil KB [40]	2019	Yes/1997/20	No	Possible resistances.
Côte d’Ivoire	Vuong PN [33]	1992	Yes/NA/NA	No	The study measures the effect of Ivermectin 3 days after administration.
	Soungalo T [32]	1997	Yes/SD/SD	No	Unknown reasons for not fully understanding the epidemiological significance of ongoing transmission.
	Koudou BG [61]	2018	Yes/1992/24	Yes/1975/16	Unknown reasons for not fully understanding the epidemiological significance of ongoing transmission.
Togo	Banla M [57]	2014	Yes/1985/23	No	Unknown reasons for not fully understanding the epidemiological significance of ongoing transmission.
	Komlan K [63]	2018	Yes/1998/24	Yes/1976/19	Unknown reasons for not fully understanding the epidemiological significance of ongoing transmission.
Liberia	Taylor HR [43]	1990	Yes/1987/3	No	Ivermectin effective drug against onchocerciasis.
	Pacque M [42]	1991	Yes/1987/3	No	Good efficacy, safety and acceptability of Ivermectin as a suitable drug for the treatment of onchocerciasis.
Democratic Republic of Congo	Makenga Bof JCM [35]	2018	Yes/2001/13	No	Side effects, insecurity and geographical inaccessibility.
	Makenga Bof JCM [34]	2019	Yes/2003/14	No	Serious side effects.
Tanzania	Paulin HN [55]	2017	Yes/2000/15	No	Failure to carry out a full evaluation, despite the good results obtained.
	Hendy A [56]	2018	Yes/1997/19	No	Unknown reasons for not fully understanding the epidemiological significance of ongoing transmission.
Central African Republic	Yaya G [31]	2014	Yes/1990/20	No	Unknown reasons for not fully understanding the epidemiological significance of ongoing transmission.
Burundi	Newell ED [16]	1997	Yes/1991/4	No	Low coverage.
South Sudan	Raimon S [68]	2021	No	Yes/2019	Search for strategies to reduce onchocerciasis transmission.

ND: No data.

**Table 6:** Type of assessment and results in countries where transmission has not been interrupted. The countries are listed in the table according to the number of articles published, from highest to lowest.

Country	Author	Year of Publication	Year of Evaluation	What Kind of Evaluation	Population Assessed	Result of the Evaluation
Cameroon	Gardon J [26]	1997	ND	Assessment of the incidence of serious events in an area where onchocerciasis and loiasis are both endemic	> or =15 years of 17,877 people	The relative risk of developing marked or serious reactions was significantly higher when the L loa load exceeded 8000 microfilariae/mL.
	Boussinesq M [5]	1997	1995	Parasitological	ND	The study provides available data on the endemicity of *Loa loa* and maps showing the prevalence of *Loa loa* microfilaridermia throughout the endemic area of infection are presented.
	Seidenfaden R [27]	2001	1996 and 1998	Entomological	121,993 flies	Pre-intervention the parous rate was 61.2% of the dissected flies. When measured at the same season after 10 years of ivermectin treatment it was 61.5%, indicating that the age structure of the fly population had not changed over the period.
				Parasitological	ND	The prevalence of human onchocerciasis in the village populations of Sora Mboum and Reh decreased from 80% in 1986/1987 to 63% in 1992/1993 and to 23% in 1996/1997.
	Twum Danso N [24]	2003	1998 and 1999	Incidence of serious adverse events (SAEs) after mass treatment withivermectin in areas co-endemic for loiasis and onchocerciasis	The study population was defined as the total number ofpeople treated with ivermectin under the auspices ofonchocerciasis mass treatment programmes in Central, Littoral and West Provinces of Cameroon.	The corresponding rates for the most severely affected district within Central Province (Okola) were 10.5 per 10,000 and 9.2 per 10,000, respectively. Symptoms began within the first 24–48 h of ivermectin administration but there was a delay of approximately 48–84 h in seeking help after the onset of symptoms. First-time exposure to ivermectin was associated with development of PLERM.
	Pion SD [30]	2004	2002	Parasitological	273 children between 5 and 9 years old	mf prevalence down from 74.6% in 1991 to 46.3% in 2002
	Richards FO [28]	2011	2008 and 2010	Parasitological	775 people	Adults had mf and nodule rates of 4.8% and 13.5%, respectively, and 5.5% had mf in the anterior chamber of the eye. Strong evidence of ongoing transmission was found in one health district, where despite 17 years of annual treatments, the annual transmission potential was 543 L3/person per year; additionally, children under 10 years of age had a 2.6% mf prevalence.
				Eyepiece	775 people	5.5% mf in anterior chamber of eye.
			2008 and 2009	Entomological	12,107 flies	Annual transmission 543 L3/person/year.
	Kamga HL [18]	2011	ND	Parasitological	404 persons	Prevalence of mf at 3.5% and of nodules 3.7%.
	Katabarwa MN [25]	2013	2005, 2006, and 2011	ND	Parasitology in adults and children	Prevalence of mf in adults decreased from 68.7% to 11.4% and of nodules from 65.9% to 12.1%. In children 29.2% to 8.9%.
			2011 and 2012	Entomological	Flies	Flies with infectivity rates of 0.19% to 0.18%.
	Wanji S [29]	2015	2011 and 2012	Entomological	ND	Transmission potentials as high as 1211.7 infective larvae/person/month were found in some of the study communities. Entomological indices followed the same trend as the parasitological indices in the three river basins with the Meme having the highest values.
				Parasitological	2797 people	The highest mean microfilaria (mf) prevalence was recorded in the Meme (52.7%), followed by Mungo (41.0%) and Manyu drainage basin (33.0%). The same trend was seen with nodule prevalence between the drainage basins. (23/39) communities (among which 13 in the Meme) still had mf prevalence above 40%.
	Eisenbarth A [17]	2016	2009–2013	Entomological	39,082 flies	Presence of infective larvae.
	Kamga GR [19]	2016	2015	Parasitological	754 people	Decrease in prevalence of mf/ss from 20.40–28.50 in 1999 to 0.48–1.74 in 2015.
	Kamgno J [23]	2017	2015	Parasitological	16,259 people	The LoaScope-based test-and-not-treat strategy enabled the reimplementation of community-wide ivermectin distribution in a heretofore “off limits” health district in Cameroon and is a potentially practical approach to larger-scale ivermectin treatment for lymphatic filariasis and onchocerciasis in areas where *L. loa* infection is endemic.
	Siewe Fodjo JN [20]	2018	2017 and 2018	Serological	1525 people	In total, 47.6% of children aged 7 to 10 years included in the study had OV16 antibodies.
	Boullé C [21]	2019	2017	Serological	A total of 307 children aged 7–10 years were examined (121 in Bayomen, 85 in Ngongol and 101 in Nyamongo).	The proportions of Ov16-positive children did not differ significantly between the three villages (55.4, 42.4 and 46.5%, respectively; *p* = 0.156). The values tended to decrease with age (54.2, 51.9, 46.8 and 41.0% in children aged 7, 8, 9 and 10 years, respectively), the difference being not significant (*p* = 0.309).
	Abong RA [22]	2021	ND	Entomological	22,274 flies	Same trends before and after mass distribution of Ivermectin.
Sierra Leone	Whitworth JAG [50]	1991	ND	Eyepiece	586 people:	The Ivermectin group had less anterior eye segment disease than the placebo group.
					296 received Ivermectin and 272 placebo	
	Njoo FL [48]	1992	1989	Parasitological	87 people	Significant decrease in microfilariae but 44% of patients were still (+)
	Bissan Y [72]	1995	1994	Entomological	1844 flies	Reduction of the average sting rate from 59.9% in 1988 to 1.0% in 1994.
	Chavasse DC [52]	1995	1987–1990	Entomological	64,215 flies caught and 17,460 analyzed	High annual sting rates (100,000 per year) and potential transmission (5000 larvae per year).
	Whitworth JAG [49]	1996	1994	Parasitological	948 people	mf prevalence of 16% 6 months after 4 doses per year and 13% after 10 doses every 6 months.
	Kläger SL [51]	2007	ND	Parasitological	77 persons	Significant reduction in the proportion of female worms found alive in the nodules and a reduction in reproductivity of 90% or more, but the majority of worms were still alive and fertile.
	Koroma JB [10]	2018	2010	Parasitological	56,521 people	Overall microfilariae prevalence decreased by 60.26% and overall average microfilariae density by 71.29%.
Ghana	Alley EN [39]	1994	1992	Parasitological	268 persons	Overall reduction in mf burden observed between baseline and 1 year after treatment was 90% in the study population and 93% in the cohort that received 5 rounds of treatment.
	Osei- Atweneboana MY [41]	2007	2004 and 2005	Parasitological	2501 people	In total, 487 (19%) of 2501 were (+). The prevalence of mf and the burden of mf in the treated community ranged from 2.2% to 51.8% and 0.06 to 2.85 mf/biopsy.
	Wilson MD [71]	2012	ND	Entomological	Testing types of repellents	NO MAS was more effective, with 5 h of effectiveness versus DEET with 1 h of effectiveness.
	Lamberton PHL [62]	2015	ND	Entomological	16,443 flies	Total counts were 463 (0.03 larvae/fly), 97 (0.6%) infected flies, and 62 (0.4%) infective flies. In the abdominal examination, 258 flies were (+) to *O. volvulus.*
	Garms R [74]	2015	2006 and 2013/2014	Entomological	ND	In 2006, there was no noticeable change in transmission intensities, only a slight reduction in fly infection rates. In 2013 and 2014, the vector unexpectedly disappeared, and transmission ceased. It is thought to have been due to the intensive mining activity in the Ofin and Pra rivers.
	Agyemang ANO [37]	2018	2006–2013	Evaluation of the CDTI Programme	1139 persons	Compliance rates decreased from 36% in 2006 to 21% in 2013. Factors affecting compliance included fear of unpleasant side effects (pruritus and oedema), which decreased from 36% to 21% for the same period. Lack of awareness of CDTI sharply increased from 12% to 46% for the same period. Participants believed that treatments were no longer necessary due to the absence of vectors observed in 2013.
	Otabil KB [40]	2019	2017	Parasitological	114 persons	Prevalence of microfilaria in the Tanfiano, Senya, Kokompe communities were 13.2, 2.4, and 2.9%, with nodule prevalence being 5.3, 4.9 and 14.3% respectively.
Côte d’Ivoire	Vuong PN [33]	1992	3 days after treatment	Parasitological	30 persons	This dose causes a nearly complete disappearance of the intralymphatic microfilariae and, surprisingly, of the “extra-vascular” ones.
	Soungalo T [32]	1997	6 months after treatment	Parasitological	1759 people	The prevalence rate of microfilaria dropped from 60.1 to 33.2%. The community microfilarial load was reduced from 29.7 to 5.6 microfilariae per skin snip.
	Koudou BG [61]	2018	2014 and 2016	Parasitological	ND	Reduction of the overall prevalence of mf by 68.1% from 43.5% to 13.9%. Decrease of mf burden in 7 out of 8 communities by 95.2% from 9.24 to 0.44 mf/biopsy.
Togo	Banla M [57]	2014	ND	Eyepiece	82 people	Elimination and prevention of mf migration in the anterior chamber of the eye and cornea. All other ocular lesions resolved and others followed.
	Komlan K [63]	2018	2015	Parasitological	1455 persons	Prevalence of mf: children aged 1–10 years 2.3 and in adults: 5.1 and 13.3%.
				Serological	14,455	OV16 (+) all ages: 48 and 34.4% and children 29.1 and 14.9% and in the Keran Mô and Oti River basins: 51.7, 23.5, and 12.7% in children.
				Entomological	ND	Infection rates in *S. damnosum* 1, 0.5, 0.1 and 0.2.
				Eyepiece	1455	Eye lesions were observed in children and young adults.
Liberia	Taylor HR [43]	1990	1987 & 1989	Parasitological	ND	prevalence of infection in 5-year-olds was reduced by 21% and the annual incidence in uninfected children was reduced by 35%.
	Pacque M [42]	1991	ND	Parasitological	ND	There was an 84% reduction in mf density in adults 2 years after treatment.
Democratic Republicof Congo	Makenga Bof JCM [35]	2018	2016	Evaluation of the CDTI Programme	21 projects implemented to control the disease were considered, representing the coverage of 42,778 endemic villages.	In total, 15,700 endemic villages were not treated through an annual CDTI with Mectizan, i.e., 36.7%. The population at risk totalled 29,712,381 individuals and 7,681,995 of them were not treated, i.e., 25.9%. Factors independently associated with non-treatment were the fear of serious side effects, supply impaired by insecurity and geographical inaccessibility.
	Makenga Bof JCM [34]	2019	2003 and 2017	To assess the frequency of post-CDTI SAE as well as factors associated with the occurrence of SAE.	15 projects out of 22 projects implemented in the country	The total average population treated was around 15,552,588 among, of which 945 cases of SAE were registered in the DR Congo, i.e., 6 cases of SAE per 100,000 persons treated per year. In total, 55 deaths related to post-CDTI SAE were recorded, which represents 5.8% of all cases of SAE. Non-neurological SAE were dominated by severe headaches (74.8%), myalgia (64.0%) and arthralgia (62.7%). Neurological SAE were mainly coma (94.1%), motor deficit (75.4%) and palpebral subconjunctival haemorrhages (38.8%).
Tanzania	Paulin HN [55]	2017	2015	Serological	948 people	OV16 (+) in 38 persons 5.59%, with 1 (0.5), 1 (0.4) and 2 (0.8%) in children <15 years.
	Hendy A [56]	2018	ND	Entomological	12,452 flies	In total, 0.57% of infective parasites carried stage L3 *S. damnosum*.
Central African Republic	Yaya G [31]	2014	2010	Parasitological	393 people > 5 years	Reduction: mf Prevalence from 89 to 19%; mean mf density from 54 to 0.7 mf/biopsy.
Burundi	Newell ED [16]	1997	1994	Parasitological	151 people	Reduction in prevalence of infection in untreated 4–5-year-olds from 60.0 to 23.7%, microfilarial load from 3.0 to 0.4 mf/biopsy.
South Sudan	Raimon S [68]	2021	2019	Entomological	400 flies dissected	*S. damnosum* s.l. close to the Maridi dam spillway decreased by >90% post-“slash and clear” for more than six months. Twelve months after the “slash and clear” intervention, the reduction in biting rates was still at <50% Parity rates reduced from 13% pre-“slash and clear” to 5.6% post-“slash and clear”.

ND: No data; mf: microfilariae; OV16: Onchocerca polymorphism diagnostic tests based on antibody detection by ELISA OV16; SAE: severe adverse effects; PLERM: encephalopathy temporally related to Mectizan treatment.

#### 3.2.3. Country Where Onchocerciasis Transmission Was Thought to Be Interrupted but Resurged After Cessation of Interventions

##### Burkina Faso

In Burkina Faso, onchocerciasis ceased to be a public health problem when the OCP in West Africa ended at the end of 2002, after 14 years of vector control strategies in place, when the level of endemicity began to decrease significantly after 4 years of vector control and became very low in 1989. This situation has been maintained without any vector control activity or chemotherapy, and no incidence of any new cases has been detected. An eye assessment carried out in 2000 by Hougard JM et al. showed only scarring of eye lesions in the examined population [70]. However, an epidemiological assessment taken place between November 2010 and February 2011 showed a resurgence of infection in the Cascades region of Burkina Faso. The level of reduction in microfilariae loads shown in the 2012 evaluation suggests that ivermectin was still effective against the recrudescent *O. volvulus* population. This finding was made at the time when ivermectin, a drug recommended for the treatment of both onchocerciasis and lymphatic filariasis, had been distributed in this area since 2004 for elimination of lymphatic filariais [73]. The authors recommended further research to determine the causes of onchocerciasis recrudescence in this area [70,73].

### 3.3. Quality Assessment

Overall, the studies were of medium or good quality (average of 11.1 points). The main reasons for scoring lower were not properly identifying the sources for data or the study period (Supplementary Materials). The most frequent limitations identified by the studies’ authors were concerns about the study and/or model accuracy [10,20,22,24,28,30,34,35,44,56,60,61,67,71] and the limited data availability [21,23,24,44,46,52,62,64].

## 4. Discussion

This literature review was carried out to evaluate the epidemiological impact of different onchocerciasis elimination interventions in hyper- and meso-endemic areas of countries in Africa. Our results showed a positive epidemiological impact on the fight against onchocerciasis, albeit with variability by country and region.

In eight countries (Uganda, Nigeria, Mali, Senegal, Sudan, Equatorial Guinea, Ethiopia, and Burkina Faso), onchocerciasis transmission has been interrupted in some zones or areas, although in Burkina Faso the disease reappeared several years after it was thought to have disappeared, following 14 years of vector control. The results of this intervention led to the conclusion that 14 years of vector control may achieve long-term elimination of onchocerciasis, even in the absence of chemotherapy [70,73]. This is confirmed by epidemiological information published by WHO in its 2021 report, showing these countries’ achievements in terms of basic reproductive rate below 1 [80]. Vector control activities have also shown to be useful for outbreak control, as demonstrated in Uganda, where the disease resurged in Nyagak-Bondo [81]. The success of these measures allowed this country to progress towards the post-treatment surveillance phase prior to the verification of disease elimination.

Senegal is also achieving notable milestones. Following the required evaluations, treatment was stopped in this country in 2022 and the 3-year post-treatment surveillance phase was initiated. Senegal will be the second country in Africa to submit a verification dossier to WHO, after Niger, which became the first country in Africa to submit it to verify elimination of transmission in 2023 [8]. In these countries, the main intervention implemented was CDTI. Of the eight countries that achieved interruption of onchocerciasis transmission in some of their foci, five have used mass distribution of ivermectin as the main strategy, while the rest implemented mix measures. This shows that ivermectin remains the appropriate drug for the treatment of onchocerciasis—it has been used since the 1980s, when it proved to be a potent microfilaricide against *O. volvulus*—and that mass treatment of an affected population can reduce transmission of the parasite [82]. This finding coincides with the good results demonstrated with CDTI in Latin America, where it has succeeded in eliminating onchocerciasis in Colombia [83], Ecuador [84], Guatemala, and Mexico [85].

In determining how long CDTI should be taken to achieve interruption of onchocerciasis, transmission has been one of the main issues discussed since its implementation. Some studies have shown that the duration depends on pre-control endemicity and treatment coverage, estimating that in areas with medium and high levels of endemicity and with treatment coverage between 65% and 80%, CDTI should last between 25 years and 40 years, respectively [78]. Other studies based on mathematical modelling have estimated that a 25-year duration of continuous long-term treatment with ivermectin can achieve suppression and interruption of *O. volvulus* transmission. Although this model is not exhaustive, it showed that variation in factors such as baseline infection rate, endemicity (transmission intensity), and numerous programmatic issues (e.g., treatment coverage and adherence) could reduce the intervention between 5 and 15 years [86]. In our study, we observed that beyond the duration of the intervention, other key factors to success must exist, as there are countries where it has been administered for a long interval (e.g., Ghana [37]) without evidence for transmission interruption.

While ivermectin has proved to be effective in the control of onchocerciasis and, even as the sole control intervention, to be able to interrupt onchocerciasis transmission, a study conducted by *Ndyomugyeny* et al. in Uganda, showed that ivermectin treatment in parallel with vector elimination has a greater impact on onchocerciasis-linked dermatitis and microfilariae carrier rates than ivermectin treatment alone [78]. In fact, three of the eight countries that have managed to interrupt onchocerciasis transmission in some of their foci (Uganda, Equatorial Guinea, and Burkina Faso) have implemented both control measures. While Uganda has managed to eliminate the onchocerciasis disease in 15 of its foci, Equatorial Guinea is about to start the ESPEN evaluation of the current onchocerciasis situation on Bioko Island, where ivermectin distribution was stopped in 2012 in the urban areas of Malabo and in 2016 in the rest of Bioko Island [67].

Despite the significant advancements brought about by the arrival of ivermectin in the fight against onchocerciasis, it has not proven to be a panacea in the treatment of onchocerciasis. According to the results of our study, 10 countries have not managed to interrupt the transmission of onchocerciasis despite several years of CDTI. We highlight the cases of Cameroon and Ghana, which, having carried out mass distribution of ivermectin for 13 to 24 years in the case of Ghana [37,40,62,74] and for 15 to 25 years in the case of Cameroon [17,19,21,25], have not managed to discontinue the transmission of the disease. Among the reasons for failing to meet the targets, a study conducted by Agyemang et al. in Ghana identified issues such as declining treatment adherence rates, fear of unpleasant side effects or opposition to CDTI [37].

In Cameroon, a study conducted by Kamga et al. highlighted the need for research on treatment coverage and an assessment of socio-anthropological and entomological factors to better understand the reasons behind the failure to achieve the expected targets in the communities of Bafia and Yabassi [19]. Other potential reasons included the occurrence of severe side effects following ivermectin treatment in communities co-endemic for onchocerciasis and loiasis [23] as well as the presence of abundant *Simulium* populations during both the dry and rainy seasons [22].

Other factors influencing the success of these interventions may include disease perception, perceived benefits of treatment, the interest and commitment of local authorities, and the occurrence or absence of post-treatment side effects [11]. Additionally, Agyemang et al. reported that non-compliance with treatment was primarily attributed to side effects, a lack of disease awareness among immigrants, and insufficient financial incentives for community distributors of ivermectin. Effective treatment adherence appeared to depend on highly motivated and widely accepted community ivermectin distributors [37].

Another reason highlighted by the studies conducted in Ghana by Osei-Atweneboana et al. [41] and Otabil et al. [40] is the potential emergence of resistance in adult parasite populations. Although ivermectin remains effective against *O. volvulus*, the possibility of ivermectin resistance is a critical concern that must be addressed to achieve the elimination of this disease. A high rate of microfilariae repopulating the skin could maintain parasite transmission, and in the presence of ivermectin-resistant *O. volvulus*, this could lead to a resurgence of the disease [40,41].

Nine studies assessed mixed methods [61,62,63,64,65,66,67,68,69]. Supplementing CDTI with vector control can significantly enhance efforts toward onchocerciasis elimination, alongside strategies such as vegetation clearance [87]. Controlling blackflies reduces the population of potential onchocerciasis vectors, thus hindering disease transmission [82].

Moreover, the “One Health” approach is becoming increasingly important in the fight against disease. This is demonstrated by a study in South Sudan, where environmental clean-up efforts, including slash-and-clear activities at the Maridi dam spillway were undertaken [68].

To address the challenges described above, especially those related to high endemicity or coendemicity for loasis, as well as treatment of patients with high *Loa loa* microfilaraemia or previous adverse reactions to ivermectin, new treatment strategies are being explored. Among these, moxidectin has emerged as a promising new alternative that may accelerate progress toward onchocerciasis elimination [88,89,90]. Furthermore, close epidemiological surveillance is crucial following the elimination of transmission to prevent disease recrudescence.

The diagnostic methods used to assess the impact of onchocerciasis control measures varied throughout the study period. They were mostly parasitological (search for microfilariae on the skin) and different from the current WHO recommendations for the verification of the interruption of onchocerciasis transmission. However, in all studies that have demonstrated interruption of onchocerciasis transmission, the methods recommended by the WHO in its new guidelines were used: entomological evaluation using PCR of O-150 and serological evaluation using Ov 16 [91].

## 5. Conclusions

### 5.1. Limitations

The limitations of our study are similar to those of any literature review study: on one hand, the possible lack of information on several onchocerciasis-endemic countries that are implementing interventions to fight onchocerciasis. It should be considered that the majority of studies cited in this article are likely biased toward heavily endemic areas, where OCP and APOC were first implemented. Moreover, a positive result bias is also likely, meaning that evaluations which fail to demonstrate transmission elimination are less likely to be published than assessments that demonstrate successful elimination

Regarding methodological bias, the limitation of the databases we used to search for scientific information, including limited access to full texts of some important publications should be considered. The average quality of the selected articles was good. However, a minority were of low quality due to incorrect descriptions of methodology, which affects the validity of our results. Additionally, we only searched for scientific publications in peer-reviewed journals. Therefore, we did not include grey literature or official national or international reports. However, we believe that a future review of these sources could provide valuable insights into onchocerciasis control measures across the African continent.

### 5.2. Conclusion and Challenges for the Future

The results of our research indicate that the interventions implemented for the control and elimination of onchocerciasis have been successful and are on track for some regions. However, we must also acknowledge the challenges faced by certain countries that, despite several years of mass distribution of ivermectin, have not achieved their goal of interrupting onchocerciasis transmission due to various reasons. These challenges should be considered by the ESPEN programme.

To move towards the elimination of onchocerciasis and achieve the 2030 targets, several actions should be prioritised. Firstly, mapping of onchocerciasis in the African region must be completed to identify remaining untreated areas of active transmission that require intervention, regardless of the level of endemicity. Secondly, areas with onchocerciasis and loasis co-endemicity in different African countries should be mapped. Thirdly, new and safe implementation strategies not only addressing loasis co-endemicity but also ecologically sound and sustainable vector control strategies must be developed to avoid serious adverse events related to loasis co-endemicity [92]. In this regard, a comprehensive approach that includes loasis co-endemicity is recommended. Fourthly, research aimed at discovering new antifilarial agents to increase the efficacy of ivermectin and address the potential emergence of ivermectin resistance must be prioritised. Finally, cross-border coordination in the fight against onchocerciasis, along with enhanced surveillance activities and mass ivermectin distribution under community guidelines must be strengthened.

## Figures and Tables

**Table 1:** Type of intervention resulting from published articles by countries. (NA: Not applicable).

Country	CDTI	Mixed	Vector Only
Burkina Faso	Nikième AS [73]	NA	Hougard JM [70]
Burundi	Newell ED [16]	NA	NA
Cameroon	Eisenbarth A [17], Kamga HL [18], Kamga GR [19], Siewe Fodjo JN [20], Boullé C [21], Abong RA [22], Kamgno J [23], Twum Danso N [24], Katabarwa MN [25], Gardon J [26], Seidenfaden R [27], Katabarwa MN [28], Boussinesq M [5], Wanji S [29], Pion SD [30]	NA	NA
Central African Republic	Yaya G [31]	NA	NA
Côte d’Ivoire	Soungalo T [32], Vuong PN [33]	Koudou BG [61]	NA
Democratic Republic of Congo	Makenga Bof JCM [34], Makenga Bof JCM [35]	NA	NA
Equatorial Guinea	NA	Herrador Z [67]	Mas J [11]
Ethiopia	Katabarwa MN [36]	NA	NA
Ghana	Agyemang ANO [37],	Lamberton PHL [62]	Wilson MD [71]
	Garms R [74]		
	Alley EN [39]		
	Otabil KB [40]		
	Osei-Atweneboana MY [41]		
Liberia	Pacque M [42]	NA	NA
	Taylor HR [43]		
Mali	Traore O [44]	NA	NA
	Diawara L [45]		
Nigeria	Richards FO [60]	NA	NA
	Tekle AH [46]		
	Onah IE [47]		
Senegal	Traore O [44]	NA	NA
	Diawara L [45]		
Sierra Leone	Koroma JB [10]	NA	Bissan Y [72]
	Njoo FL [48]		
	Whitworth JAG [49]		
	Whitworth JAG [50]		
	Kläger SL [51]		
	Chavasse DC [52]		
South Sudan	NA	Raimon S [68]	NA
Sudan	Higazi TB [53]	NA	NA
	Zarroug IM [54]		
	Katabarwa MN [36]		
Tanzania	Paulin HN [55]	NA	NA
	Hendy A [56]		
Togo	Banla M [57]	Komlan K [63]	NA
Uganda	Katabarwa MN [59]	Katabarwa MN [65]	Garms R [38]
	Luroni LT [58]	Lakwo T [64]	
		Katabarwa MN [66]	
		Lakwo TL [69]	

**Table 2:** Minimum and maximum years of CDTI per country.

	Years of CDTI (Until Evaluation)	
Country	Min	Max
Uganda	14	24
Nigeria	8	26
Mali	15	17
Senegal	18	23
Sudan	13	14
Equatorial Guinea	22	26
Ethiopia	14	16
Average	15	21
Standard deviation	4.34	5.05

## Data Availability

The dataset supporting the conclusions of this article is included within the article (and its additional files in the Supplementary Materials).

## Supplementary Materials

The following supporting information can be downloaded at: https://www.mdpi.com/article/10.3390/tropicalmed10010007/s1, Table S1: Countries that have successfully interrupted transmission of onchocerciasis in certain areas. The countries are listed in the table according to the number of articles published, from highest to lowest. (ND: No data); Table S2. Type of assessment and results in countries that have successfully interrupted transmission of onchocerciasis in certain areas. The countries are listed in the table according to the number of articles published, from highest to lowest; Table S3: Countries where transmission has not been interrupted. The countries are listed in the table according to the number of articles published, from highest to lowest; Table S4: Type of assessment and results in countries where transmission has not been interrupted. The countries are listed in the table according to the number of articles published, from highest to lowest; Table S5: Country that succeeded in interrupting onchocerciasis transmission and has seen a reoccurrence (Burkina Faso). Type of assessment and results in countries that succeeded in interrupting onchocerciasis transmission and has seen a reoccurrence (ND: No data). PRISMA checklist.
