# Supplementary material for: The Impact of Onchocerciasis Elimination Measures in Africa: A Systematic Review
Source: Trop Med Infect Dis. 2024 Dec 26;10(1):7. doi: 10.3390/tropicalmed10010007 (PMC11768683; doi:10.3390/tropicalmed10010007)
Supplement: Supplementary file 1 [file tropicalmed-10-00007-s001.zip › tropicalmed-3327067-supplementary.pdf]

Table S1. Countries that have successfully interrupted transmission of onchocerciasis in certain areas. The countries are listed in the table according to the number of articles published, from highest to lowest. (ND: No data)

	**Applied intervention**									
	**CDTI**	**Start date**	**Duration in years**	**Control Vector**	**Start date**	**Duration in years**	**Transmission interrupted in some regions**	**Regions with transmission interruption**	**Year of publication**	**Author**
	**Yes/No**			**Yes/No**						
Uganda	Yes	2008	5	Yes	2009		Yes	Wambabya - Rwamarongo	2020	Katabarwa MN [76]
	Yes	1991	19	Yes	2003	3	Yes	Kashoya - Kitomi	2017	Lakwo T [69]

	Yes	1993	17	No	ND	ND	Yes	Imaramagambo	2016	Katabarwa MN [59]
	Yes	1994	17	Yes	2008	1	Yes	Mount Elgon	2014	Katabarwa MN [66]
	No	ND	ND	Yes	1951	22	Yes	Nile Victoria	2020	Katabarwa MN [65]
	Yes	1991	19	Yes	1995	ND	Yes	Itwara	2013	Lakwo TL [64]
	Yes	1993	14	No	ND	ND	Yes	Wadelai	2012	Katabarwa MN [69]
	Yes	1995	17	Yes	2002	ND	Yes	Mpamba Kusi	2015	Lakwo TL [75]
	Yes	1993	20	No		ND	Yes	Obongi	2017	Luroni LT [58]
	No	ND	ND	Yes	1995	8	Yes	Itwara	2009	Garms R [38]
Nigeria	Yes	1992	26	No	ND	ND	Yes	Plateau and Nasarawa	2020	Richards FO [60]
	Yes	1991	17	No	ND	ND	Yes	Kaduna	2012	Tekle AH [46]

	Yes	1996	15	No	ND	ND	Yes	Adani	2020	Onah IE [47]
Mali	Yes	1998	17	No	ND	ND	Yes	Bakoye and Faleme	2012	Traore O [44]
	Yes	1998	17	No	ND	ND	Yes	Bakoye and Faleme	2009	Diawara L [45]
Senegal	Yes	1998	17	No	ND	ND	Yes	Gambia and Faleme	2012	Traore MO [44]
	Yes	1998	17	No	ND	ND	Yes	Gambia and Faleme	2009	Diawara L [45]
Sudan	Yes	1998	14	No	ND	ND	Yes	Abu Hamed	2013	Higazi TB [53]
	Yes	1998	14	No	ND	ND	Yes	Abu Hamed	2016	Zarroug IM [54]
	Yes	2007	11	No	ND	ND	Yes	Galabat	2020	Katabarwa MN [36]
Equatorial Guinea	Yes	1990	26	Yes	2001	4	Yes	Bioko Island	2006	Mas J [11]
	No	ND	ND	Yes	2001	4	Yes	Bioko Island	2009	Herrador Z [67]
Ethiopia	Yes	2003	14	No	ND	ND	Yes	Metema	2020	Katabarwa MN [36]

Table S2. Type of assessment and results in countries that have successfully interrupted transmission of onchocerciasis in certain areas. The countries are listed in the table according to the number of articles published, from highest to lowest.

	Year of evaluation	What kind of evaluation	Population assessed	Result of the evaluation	Author	Year
Uganda	2016	Entomological	1,0578 crabs	0 crabs infested<br>0 fly caught	Katabarwa MN [76]	2020
	2013	Serological	2,978 minors	1 positive only		
	2017	Serological	3,079 minors	0 positive		
	2009 & 2013	Entomological	Crabs and flies	Crab infestation reduced from 59 % to 0 %.<br>Fly Catch from 5,334 to **0**	Lakwo T [69]	2017
	2010	Serological	1,362 children	11 children (+) OV16		
	2012	Serological	3,246 children	5 children (+) OV16 and 4 children out of 5 (-) PCR		
	2004 & 2013	Parasitological	Number of microfilariae	From 85 % to 62 % in 2004 to 0.5 % 2013		
	2015	Entomological	Fly Catching	0 flies caught since 2007	Katabarwa MN [59]	2016
	2009	Serological	3,051 children	1 (+)	Katabarwa MN [66]	2014
	2015	Entomological	Crabs and flies	Infestation reduced from 41.9% 2007 to 0% and 0 fly bites		
	ND	Entomological	854 flies	0 infected	Katabarwa MN [65]	2020
	ND	Serological	2,953 children	All (-) OV16		

	2010 & 2012	Serological	3,316 children	2 (+) OV16. In 2012, 21 months later the 2 children were (-) OV16.	Lakwo TL [64]	2013
		Parasitological	688 skin snips	No evidence of any microfilaria		
	2009	Parasitological	500 people	0 microfilariae	Katabarwa MN [75]	2012
	2009	Serological	3,011 children	1 child (+) OV16		
	2008	Entomological	14,391	0 infested	Lakwo TL [69]	2017
	2009 & 2012	Serological	3,351 <15 years and 3,407	0.6 % while in 2012 only 1 out of 3,407 (+)		
	2012	Parasitological	732 people	0,3 % of microfilariae		
	2012	Parasitological	807 people	0% microfilariae	Luroni LT [58]	
	2013	Serological	3,308 < 10 years	3 (+) OV16, by PCR all 3 (-)		
	2003	Entomological	Flies	0 flies found	Garms R [38]	2009
Nigeria	2017	Serological	6,262 < 10 years	2 children (+)	Richards FO [60]	2020
	2017	Entomological	19,056 flies	None infested		
	2008	Parasitological	3,703 > 1 year	0 % prevalence	Tekle AH [46]	2012
	2011	Entomological	548 flies	0 infested	Onah IE [47]	2020
Mali	2006 - 2011	Parasitological	29,753 people	Indicators below the elimination threshold	Traore MO [44]	2012
	2006 - 2011	Entomological	492,600 flies	Indicators below the elimination threshold		
	ND	Parasitological	17,801 people<br>2,283 people	Prevalence < 1 %.	Diawara L [45]	2009
	ND	Entomological	157,500 flies<br>123,000 flies	Infestation rate 0.5		
Senegal	2006 - 2011	Parasitological	29,753 people	Indicators below the elimination threshold	Traore MO [44]	2012
	2006 - 2011	Entomological	492,600 flies	Indicators below the elimination threshold		

	ND	Parasitological	17,801 people<br>2,283 people	Prevalence < 1 %.	Diawara L [45]	2009
	ND	Entomological	157,500 flies<br>123,000 flies	Infestation rate 0.5		
Sudan	2011	Parasitological	536 persons	Skin microfilariae were absent	Higazi TB [53]	2013
	2011	Serological	6,756 < 10 years	No evidence of *O. volvulus* antibodies were found		
	2011	Entomological	17,536 flies	O-150 pool screening showed no parasite DNA in 17,537 black flies (95% CI UL 0.023)		
	2015	Serological	5,266 children	Only one Ov16 seropositive child (0.019%, 95% UCL = 0.074); whose skin snips were negative when tested by O-150 PCR assay	Zarroug IM [54]	2016
	2015	Entomological	19,266 flies	Polymerase chain reaction (PCR) screening of 19,191 flies from four sites for the O-150 parasite-specific marker found no flies carrying *O. volvulus* larvae (0%, 95% upper confidence limit [UCL] = 0.16)		
Equatorial Guinea	2016	Entomological	Fly Capture	All fly collections and larval prospections in the traditional catching and prospection sites were negative.	Herrador Z [67]	2018
	2016	Serological	7,052 <10 years	4 (+) OV16 and (-) ELISA		
	ND	Entomological	Flies	0 flies caught	Cheke R [79]	2009
Ethiopia	2014 & 2015	Serological	6,072 and 3,931 < 10 years old	8 (+) OV16 and (-) PCR	Katabarwa MN [36]	2020
	2014 & 2016	Entomological	27,583 and 9,148 flies	0.14 of infective flies		

OV16: *Onchocerca* polymorphism diagnostic tests based on antibody detection by ELISA OV16

Table S3: Countries where transmission has not been interrupted. The countries are listed in the table according to the number of articles published, from highest to lowest.

Countries	Applied intervention										
	CDTI	Start date	duration in years	Vector processing	Start date	duration in years	Transmission is maintained	It has been interrupted and has returned	Why	Year of publication	aAuthor
	Yes/No	Year		Yes/No	Year		Yes/No	Yes/No			
Cameroon	Yes	1987	25	No	ND	ND	Yes	No	This study may reveal the impact of the presence of animal filariasis on the transmission of onchocerciasis.	2016	Eisenbarth A [17]
	Yes	2004	6	No	ND	ND	Yes	No	Mid-term evaluation	2011	Kamga HL [18]
	Yes	1999	15 – 16	No	ND	ND	Yes	No	possible coverage issues and socio-anthropological considerations related to the ICTD	2016	Kamga GR [19]
	Yes	1990	+ 13	No	ND	ND	Yes	No	Sub-optimally implemented control measures	2018	Siewe Fodjo JN [20]

	Yes	1998	19	No	ND	ND	Yes	No	Unknown reasons for not fully understanding the epidemiological significance of ongoing transmission.	2019	Boullé C [21]
	Yes	1997	20	No	ND	ND	Yes	No	The possible fluvial system of the Mbam river drainage generates important breeding sites, which causes abundant *Simulium* in both the dry and rainy seasons.	2021	Abong RA [22]
	Yes	ND	ND	No	ND	ND	Yes	No	Interruption of ivermectin treatment since 1999 due to severe side effects.	2017	Kamgno J [23]
	Yes	ND	ND	No	ND	ND	Yes	No	Occurrence of serious side effects in an endemic area with *Loa loa*	2003	Twum Danso N [24]
	Yes	1996	15	No	ND	ND	Yes	No	Unknown reasons for not fully understanding the epidemiological significance of ongoing transmission.	2013	Katabarwa MN [25]
	Yes	ND	ND	No	ND	ND	Yes	No	Occurrence of severe side effects in an endemic area with *Loa loa*, after treatment	1997	Gardon J [26]
	Yes	1987	10	No	ND	ND	Yes	No	Unknown reasons for not fully understanding the epidemiological significance of ongoing transmission.	2001	Seidenfaden R [27]

	Yes	1992	17	No	ND	ND	Yes	No	Unknown reasons for not fully understanding the epidemiological significance of ongoing transmission.	2011	Katabarwa MN [28]
	Yes	1987	8	No	ND	ND	Yes	No	Unknown reasons for not fully understanding the epidemiological significance of ongoing transmission.	1997	Boussinesq M [5]
	Yes	1989	10	No	ND	ND	Yes	No	Onchocerciasis endemic area and *Loa loa* and ecological factors strongly favor onchocerciasis transmission.	2015	Wanji S [29]
	Yes	1991	4	No	ND	ND	Yes	No	Low coverage	2004	Pion SD [30]
Sierra Leone	Yes	2005	ND	No	ND	ND	Yes	ND	Unknown reasons for not fully understanding the epidemiological significance of ongoing transmission.	2018	Koroma JB [10]
	Yes	1987	2	No	ND	ND	Yes	No	Unknown reasons for not fully understanding the epidemiological significance of ongoing transmission.	1992	Njoo FL [48]
	Yes	1987	6,5	No	ND	ND	Yes	No	Unknown reasons for not fully understanding the epidemiological significance of ongoing transmission.	1996	Whitworth JAG [49]
	No	ND	ND	Yes	1989	5	Yes	No	Unknown reasons for not fully understanding the epidemiological significance of ongoing transmission.	1995	Bissan Y [72]

	Yes	SD	2	No	ND	ND	Yes	No	Unknown reasons for not fully understanding the epidemiological significance of ongoing transmission.	1991	Whitworth JAG [50]
	Yes	1987	6	No	ND	ND	Yes	No	Unknown reasons for not fully understanding the epidemiological significance of ongoing transmission.	2007	Kläger SL [51]
	Yes	1987	3	No	ND	ND	Yes	No	Unknown reasons for not fully understanding the epidemiological significance of ongoing transmission.	1995	Chavasse DC [52]
Ghana	Yes	1999	13	No	ND	ND	Yes	No	Treatment adherence rates decreased for the following reasons: fear of unpleasant side effects, lack of awareness of ITDPs, participants believed that treatments were no longer necessary due to the absence of the vectors observed in 2013, motivation of the distributors	2018	Agyemang ANO [37]
	Yes	1987	24	Yes	1975	27	Yes	No	Unknown reasons for not fully understanding the epidemiological significance of ongoing transmission.	2015	Lamberton PHL [62]
	Yes	1999	13	No	ND	ND	Yes	No	Intermittent disappearance of the vector	2015	Garms R [74]

	No	ND	ND	Yes	SD	SD	Yes	No	The effect of different types of repellents for the prevention of *Simulium damnosum* bites is tested.	2013	Wilson MD [71]
	Yes	1987	5	No	ND	ND	Yes	No	It is a part-time evaluation	1994	Alley EN [39]
	Yes	1997	20	No	ND	ND	Yes	No	Possible resistances	2019	Otabil KB [40]
	Yes	ND	6-18	No	ND	ND	Yes	No	The microfilaricide effect of Ivermectin, but that adult populations of resistant and unresponsive parasites are emerging.	2007	Osei-Atweneboana MY [41]
Côte d’Ivoire	Yes	1992	24	Yes	1975	16	Yes	No	Unknown reasons for not fully understanding the epidemiological significance of ongoing transmission.	2018	Koudou BG [61]
	Yes	SD	SD	No	ND	ND	Yes	No	Unknown reasons for not fully understanding the epidemiological significance of ongoing transmission.	1997	Soungalo T [32]
	Yes	NA	NA	No	ND	ND	Yes	No	The study measures the effect of Ivermectin 3 days after administration.	1992	Vuong PN [33]
Togo	Yes	1998	24	Yes	1976	19	Yes	No	Unknown reasons for not fully understanding the epidemiological significance of ongoing transmission.	2018	Komlan K [63]
	Yes	1985	23	No	No	**ND**	Yes	No	Unknown reasons for not fully understanding the epidemiological significance of ongoing transmission.	2014	Banla M [57]

Liberia	Yes	1987	3	No	ND	ND	Yes	No	Good efficacy, safety and acceptability of Ivermectin as a suitable drug for the treatment of onchocerciasis	1991	Pacque M [42]
	Yes	1987	3	No	ND	ND	Yes	No	Ivermectin effective drug against onchocerciasis	1990	Taylor HR [43]
Democratic Republic of Congo	Yes	2003	14	No	No	ND	Yes	No	Serious side effects	2019	Makenga Bof JCM [34]
	Yes	2001	13	No	ND	ND	Yes	No	Side effects, insecurity and geographical inaccessibility	2018	Makenga Bof JCM [35]
Tanzania	Yes	2000	15	No	ND	ND	Yes	No	Failure to carry out a full evaluation, despite the good results obtained.	2017	Paulin HN [55]
	Yes	1997	19	No	ND	ND	Yes	No	Unknown reasons for not fully understanding the epidemiological significance of ongoing transmission.	2018	Hendy A [56]
Central African Republic	Yes	1990	20	No	ND	ND	Yes	No	Unknown reasons for not fully understanding the epidemiological significance of ongoing transmission.	2014	Yaya G [31]
Burundi	Yes	1991	4	No	ND	ND	Yes	No	Low coverage	1997	Newell ED [16]

Table S4: Type of assessment and results in countries where transmission has not been interrupted. The countries are listed in the table according to the number of articles published, from highest to lowest.

	Year of evaluation	What kind of evaluation	Population assessed	Result of the evaluation	Author	Year
Cameroon	2009-2013	Entomological	39,082 flies	Presence of infective larvae	Eisenbarth A [17]	2015
	ND	Parasitological	404 persons	Prevalence of mf at 3.5 % and of nodules 3.7 %.	Kamga HL [18]	2011

	2015	Parasitological	754 people	Decrease in prevalence of mf/ss from 20.40 – 28.50 in 1999 to 0.48 – 1.74 in 2015	Kamga GR [19]	2016
	2017 y 2018	Serological	1,525 people	47.6 % of children aged 7 to 10 years included in the study had OV16 antibodies.	Siewe Fodjo JN [20]	2018
	2017	Serological	A total of 307 children aged 7–10 years were examined (121 in Bayomen, 85 in Ngongol and 101 in Nyamongo).	The proportions of Ov16-positive children did not differ significantly between the three villages (55.4, 42.4 and 46.5%, respectively; P = 0.156). The values tended to decrease with age (54.2, 51.9, 46.8 and 41.0% in chil dren aged 7, 8, 9 and 10 years, respectively), the differ ence being not significant (P = 0.309).	Boullé C [21]	2019
	ND	Entomological	22,274 flies	Same trends before and after mass distribution of Ivermectin	Abong RA [22]	2021
	Yes	Measures the occurrence of post-treatment side-effects			Kamgno J [23]	2017
	Assessment of the incidence of serious side effects after Ivermectin treatment				Twum Danso N [24]	2003

	2005, 2006 y 2011	ND	Parasitology in adults and children	Prevalence of mf in adults decreased from 68.7% to 11.4% and of nodules from 65.9% to 12.1%.<br><br>In children 29,2 % to 8,9 %.	Katabarwa MN [25]	2013
	2011 y 2012	Entomological	Flies	Flies with infectivity rates of 0.19% to 0.18%.		
	Assessment of the occurrence of side effects after Ivermectin treatment				Gardon J [26]	1997
	1996 y 1998	Entomological	121,993 flies	Potential annual transmission of infective larvae down to 40 % of pre-intervention level	Seidenfaden R [27]	2001
		Parasitological	ND	onchocerciasis prevalence in humans dropped from 80 % to 23 %.		
	2008 a 2010	Parasitological	775 people	Adults had mf and nodule rates of 4.8% and 13.5%, respectively, and 5.5% had mf in the anterior chamber of the eye. Strong evidence of ongoing transmission was found in one health district, where despite	Katabarwa MN [28]	2011

				17 years of annual treatments, the annual transmission potential was 543 L3/person per year; additionally, children under 10 years of age had a 2.6% mf prevalence.		
		Eyepiece	775 people	5.5 % mf in anterior chamber of eye		
	2008 y 2009	Entomological	12,107 flies	Annual transmission 543 L3 / person/year		
	1995	Parasitological	ND	More than 90 % reduction in prevalence	Boussinesq M [5]	1997
	2011 y 2012	Entomological	ND	ND	Wanji S [29]	2015
		Parasitological	2,797 people	The highest mean microfilaria (mf) prevalence was recorded in the Meme (52.7%), followed by Mungo (41.0%) and Manyu drainage basin (33.0%). The same trend was seen with nodule prevalence between the drainage basins. (23/39) communities (among which 13 in the Meme) still had mf prevalence above 40%.		
	2002	Parasitological	273 children between 5 and 9 years old	mf prevalence down from 74.6 % in 1991 to 46.3 % in 2002	Pion SD [30]	2004

Sierra Leone	2010	Parasitological	56,521 people	Overall microfilariae prevalence decreased by 60.26 % and overall average microfilariae density by 71.29 %.	Koroma JB [10]	2018
	1989	Parasitological	87 people	Significant decrease in microfilariae but 44 % of patients were still (+)	Njoo FL [48]	1992
	1994	Parasitological	948 people	mf prevalence of 16 % 6 months after 4 doses per year and 13 % after 10 doses every 6 months	Whitworth JAG [49]	1996
	1994	Entomological	1844 flies	Reduction of the average sting rate from 59.9% in 1988 to 1.0% in 1994.	Bissan Y [72]	1995
	ND	Eyepiece	586 people: 296 received Ivermectin and 272 placebo	The Ivermectin group had less anterior eye segment disease than the placebo group.	Whitworth JAG [50]	1991
	ND	Parasitological	77 persons	Significant reduction in the proportion of female worms found alive in the nodules and a reduction in reproductivity of 90 % or more. But the majority of worms were still alive and fertile.	Kläger SL [51]	2007

	1987 - 1990	Entomological	64,215 flies caught and 17,460 analyzed	High annual sting rates (100,000 per year) and potential transmission (5,000 larvae per year).	Chavasse DC [52]	1995
Ghana	2006 a 2013	Evaluation of the ICTD Programme	1,139 persons	Compliance rates decreased from 36% in 2006 to 21% in 2013. Factors affecting compliance included fear of unpleasant side effects (pruritus and oedema), which decreased from 36% to 21% for the same period. Lack of awareness of CDTI sharply increased from 12% to 46% for the same period. Participants believed that treatments were no longer necessary due to the absence of vectors observed in 2013.	Agyemang ANO [37]	2018
	ND	Entomological	16,443 flies	463 (0.03 larvae/fly), 97 (0.6 %) infected flies and 62 (0.4 %) infective flies. In the abdominal examination, 258 flies were (+) to *O. volvulus*	Lamberton PHL [62]	2015
	2006 y 2013/2014	Entomological	ND	In 2006, there was no noticeable change in transmission intensities, only a slight reduction in fly infection rates.	Garms R [74]	2015

				In 2013 and 2014, the vector unexpectedly disappeared, and transmission ceased. It is thought to have been due to intensive mining activity in the Ofin and Pra rivers.		
	ND	Entomological	Testing types of repellents	No MÁS was more effective with 5 hours of effectiveness versus DEET with 1 hour of effectiveness.	Wilson MD [71]	2012
	1992	Parasitological	268 persons	Overall reduction in mf burden observed between baseline and 1 year after treatment was 90% in the study population and 93% in the cohort that received 5 rounds of treatment.	Alley EN [39]	1994
	2017	Parasitological	114 persons	Prevalence of mf 13.2, 2.4 and 2.9% and of nodules 5.3, 4.9 and 14.3%.	Otabil KB [40]	2019
	2004 y 2005	Parasitological	2,501 people	487 (19%) of 2501 were (+). The prevalence of mf and the burden of mf in the treated community ranged from 2.2% to 51.8% and 0.06 to 2.85 mf/biopsy.	Osei-Atweneboana MY [41]	2007
Côte d'Ivoire	2014 y 2016	Parasitological	ND	Reduction of the overall prevalence of mf by 68.1% from 43.5% to 13.9%. Decrease	Koudou BG [61]	2018

				of mf burden in 7 out of 8 communities by 95.2 % from 9.24 to 0.44 mf /biopsy.		
	6 months after treatment	Parasitological	1,759 people	Reduction of mf prevalence from 60.1% to 33.2%. Community CMFL reduced from 29.7 to 5.6 mf / skin bio syndrome.	Soungalo T [32]	1997
	3 days after treatment	Parasitological	30 persons	Almost complete disappearance of intralymphatic and extravascular mf No	Vuong PN [33]	1992
Togo	2015	Parasitological	1,455 persons	Prevalence of mf: children aged 1-10 years 2.3 and in adults: 5.1 and 13.3%.	Komlan K [63]	2018
		Serological	14,455	OV16 (+) all ages: 48 and 34.4 % and children 29.1 and 14.9 % and in the Keran Mô and Oti River basins: 51.7, 23.5 and 12.7 % in children.		
		Entomological		Infection rates in *S. damnosum* 1, 0,5, 0,1 and 0,2		
		Eyepiece	1,455	Eye lesions were observed in children and young adults.		
	ND	Eyepiece	82 people	Elimination and prevention of mf migration in the anterior chamber of the	Banla M [57]	2014

				eye and cornea. All other ocular lesions resolved and others followed.		
Liberia	ND	Parasitological	ND	84% reduction in mf density in adults 2 years after treatment	Pacque M [42]	1991
	1987 y 1989	Parasitological	ND	prevalence of infection in 5-year-olds was reduced by 21% and the annual incidence in uninfected children was reduced by 35%.	Taylor HR [43]	1990
Democratic Republic of Congo	2003 to 2017 retrospective study	Analysis of severe adverse effects following community-based ivermectin treatment	15,552,588 persons	The total average population treated was around 15,552,588 among which 945 cases of severe adverse effects (SAE) were registered 6 cases of SAE for 100,000 persons treated per year. 55 deaths related to post-CDTI SAE were recorded, which represents 5.8% of all cases of SAE. Non-neurological SAE were dominated by severe headaches (74.8%), myalgia (64.0%) and arthralgia (62.7%). Neurological SAE were mainly coma (94.1%),	Makenga Bof JCM [34]	2019

	2016	Evaluation of the TIDC Programme	ND	Of the 42,778 endemic communities, 15,700 were not treated, 36.7%.	Makenga Bof JCM [35]	2018
Tanzania	2015	Serological	948 people	OV16 (+) in 38 persons 5.59 %, with 1 (0.5), 1 (0.4) and 2 (0.8 %) in children < 15 years.	Paulin HN [55]	2017
	ND	Entomological	12,452 flies	0.57 % of *S. damnosum* carrying infective parasites stage L3	Hendy A [56]	2018
Central African Republic	2010	Parasitological	393 people > 5 years	Reduction: mf Prevalence from 89 to 19%; mean mf density from 54 to 0.7 mf /Biopsy; community mf burden from 39 to 0.67 Mf /Biopsy and eye lesions from 28 to 2%.	Yaya G [31]	2014
Burundi	1994	Parasitological	151 people	Reduction in prevalence of infection in untreated 4–5-year-olds from 60.0 to 23.7 % CMFL from 3.0 to 0.4 mf/Biopsy	Newell ED [16]	1997

Table S5: Country that succeeded in interrupting onchocerciasis transmission and has seen a reoccurrence (Burkina Faso). Type of assessment and results in country that succeeded in interrupting onchocerciasis transmission and has seen a reoccurrence. (ND: No data).

	**Nikième AS (2021)** [73]	**Hougard JM (2001)** [70]
Applied intervention		
CDTI (Yes/No)	Yes	No
Start date	2010	ND
Duration (years)	2	ND
Vector processing	No	Yes
Start date	ND	1976
Duration (years)	ND	14
Transmission is maintained (Yes/No)	Yes	No
It has been interrupted and has returned (Yes/No)	Yes	No
Why	The reasons for the upsurge are not clear but were associated with high levels of vector infectivity.	Reason unknown
Year of evaluation	2012	2000
Type of evaluation	Parasitological	Eyepiece
Population assessed	130 people	ND
Results of the evaluation	The maximum individual mean microfilarial load was 115 mf/b (microfilariae per biopsy) in 2010 and 26 mf/b in 2012	No new eye lesions. Scarring of eye lesions in the screened population.
